# The tale of the neuroscientists and the computer: why mechanistic theory matters

**DOI:** 10.3389/fnins.2014.00349

**Published:** 2014-10-31

**Authors:** Joshua W. Brown

**Affiliations:** Psychological and Brain Sciences, Indiana UniversityBloomington, IN, USA

**Keywords:** computational modeling, methods, Theory, fMRI, EEG, Neurophysiology, Neuropsychology

## Introduction

A little over a decade ago, a biologist asked the question “Can a biologist fix a radio?” (Lazebnik, 2002). That question framed an amusing yet profound discussion of which methods are most appropriate to understand the inner workings of a system, such as a radio. For the engineer, the answer is straightforward: you trace out the transistors, resistors, capacitors etc., and then draw an electrical circuit diagram. At that point you have understood how the radio works and have sufficient information to reproduce its function. For the biologist, as Lazebnik suggests, the answer is more complicated. You first get a hundred radios, snip out one transistor in each, and observe what happens. Perhaps the radio will make a peculiar buzzing noise that is statistically significant across the population of radios, which indicates that the transistor is necessary to make the sound normal. Or perhaps we should snip out a resistor, and then homogenize it to find out the relative composition of silicon, carbon, etc. We might find that certain compositions correlate with louder volumes, for example, or that if we modify the composition, the radio volume decreases. In the end, we might draw a kind of neat box-and-arrow diagram, in which the antenna feeds to the circuit board, and the circuit board feeds to the speaker, and the microphone feeds to the recording circuit, and so on, based on these empirical studies. The only problem is that this does not actually show how the radio works, at least not in any way that would allow us to reproduce the function of the radio given the diagram. As Lazebnik argues, even though we could multiply experiments to add pieces of the diagram, we still won't really understand how the radio works. To paraphrase Feynmann, if we cannot recreate it, then perhaps we have not understood it (Hawking, 2001; Eliasmith and Trujillo, 2014).

Lazebnik's argument should not be construed to disparage biological research in general. There are abundant examples of how molecular biology has led to breakthroughs, including many if not all of the pharmaceuticals currently on the market. Likewise, research in psychology has provided countless insights that have led to useful interventions, for instance in cognitive behavioral therapy (Rothbaum et al., 2000). These are valuable ends in and of themselves. Still, are we missing greater breakthroughs by not asking the right questions that would illuminate the larger picture?

**Figure 1:**
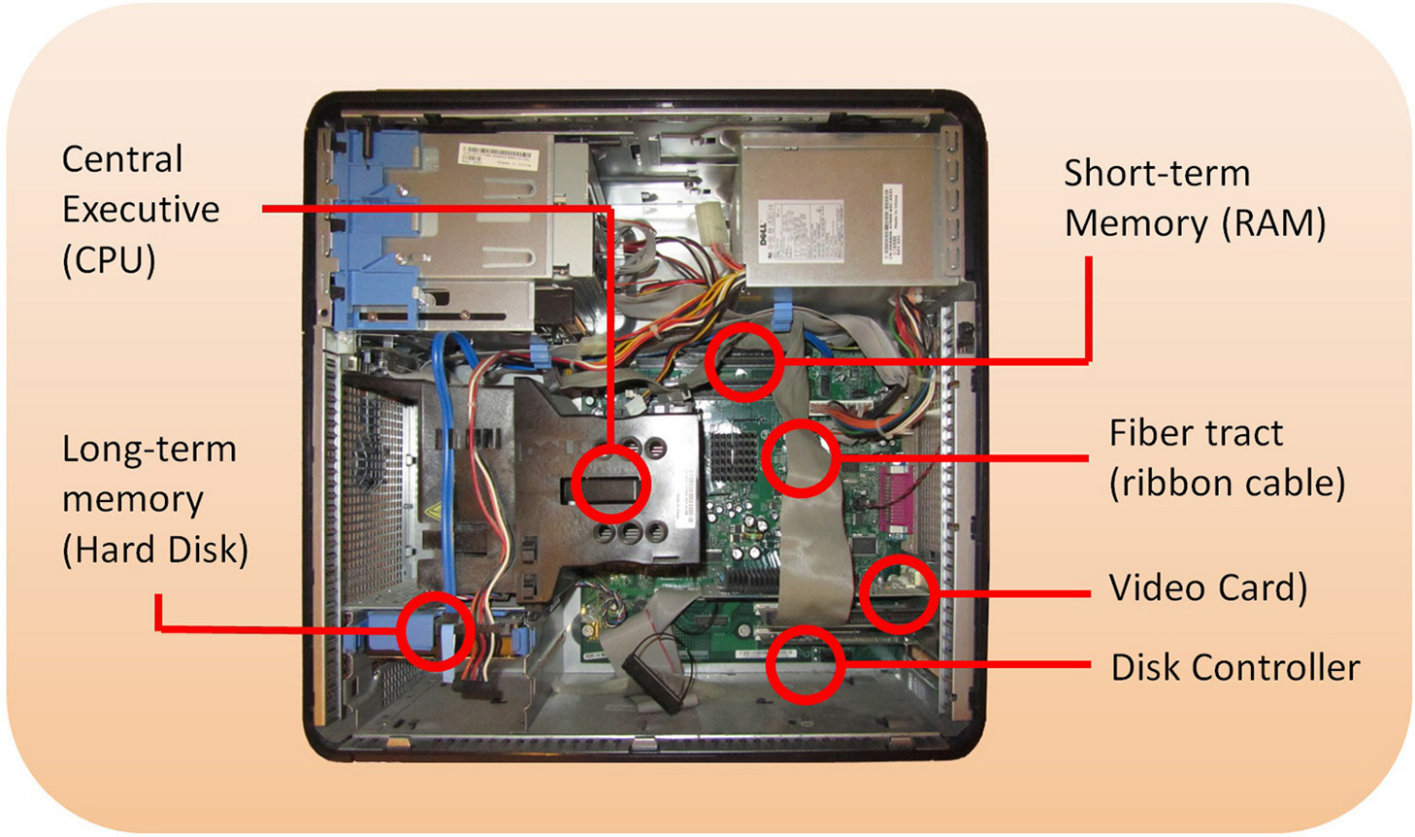
**The inside of a typical computer, showing CPU, hard disk, memory, and disk controller**.

Within the fields of systems, cognitive, and behavioral neuroscience in particular, I fear we are in danger of losing the meaning of the Question “how does it work?” As the saying goes, if you have a hammer, everything starts to look like a nail. Having been trained in engineering as well as neuroscience and psychology, I find all of the methods of these disciplines useful. Still, many researchers are especially well-trained in psychology, and so the research questions focus predominantly on understanding which brain regions carry out which psychological or cognitive functions, following the established paradigms of psychological research. This has resulted in the question being often reframed as “what brain regions are active during what psychological processes,” or the more sophisticated “what networks are active,” instead of “what mechanisms are necessary to reproduce the essential cognitive functions and activity patterns in the system.” To illustrate the significance of this difference, consider a computer (Figure 1). How does it work?

## The tale

Once upon a time, a group of neuroscientists happened upon a computer (Carandini, 2012). Not knowing how it worked, they each decided to find out how it sensed a variety of inputs and generated the sophisticated output seen on its display. The EEG researcher quickly went to work, putting an EEG cap on the motherboard and measuring voltages at various points all over it, including on the outer case for a reference point. She found that when the hard disk was accessed, the disk controller showed higher voltages on average, and especially more power in the higher frequency bands. When there was a lot of computation, a lot of activity was seen around the CPU. Furthermore, the CPU showed increased activity in a way that is time-locked to computational demands. “See here,” the researcher declared, “we now have a fairly temporally precise picture of which regions are active, and with what frequency spectra.” But has she really understood how the computer works?

Next, the enterprising physicist and cognitive neuroscientist came along. “We don't have enough spatial resolution to see inside the computer,” they said. So they developed a new imaging technique by which activity can be measured, called the Metabolic Radiation Imaging (MRI) camera, which now measures the heat (infrared) given off by each part of the computer in the course of its operations. At first, they found simply that lots of math operations lead to heat given off by certain parts of the CPU, and that memory storage involved the RAM, and that file operations engaged the hard disk. A flurry of papers followed, showing that the CPU and other areas are activated by a variety of applications such as word-processing, speech recognition, game play, display updating, storing new memories, retrieving from memory, etc.

Eventually, the MRI researchers gained a crucial insight, namely that none of these components can be understood properly in isolation; they must understand the *network*. Now the field shifts, and they begin to look at interactions among regions. Before long, a series of high profile papers emerge showing that file access does not just involve the disks. It involves a network of regions including the CPU, the RAM, the disk controller, and the disk. They know this because when they experimentally increase the file access, all of these regions show correlated increases in activity. Next, they find that the CPU is a kind of hub region, because its activity at various times correlates with activity in other regions, such as the display adapter, the disk controller, the RAM, and the USB ports, depending on what task they require the computer to perform.

Next, one of the MRI researchers has the further insight to study the computer while it is idle. He finds that there is a network involving the CPU, the memory, and the hard disk, as (unbeknownst to them) the idle computer occasionally swaps virtual memory on and off of the disk and monitors its internal temperature. This resting network is slightly different across different computers in a way that correlates with their processor speed, memory capacity, etc., and thus it is possible to predict various capacities and properties of a given computer by measuring its activity pattern when idle. Another flurry of publications results. In this way, the neuroscientists continue to refine their understanding of the network interactions among parts of the computer. They can in fact use these developments to diagnose computer problems. After studying 25 normal computers and comparing them against 25 computers with broken disk controllers, they find that the connectivity between the CPU and the disk controller is reduced in those with broken disk controllers. This allows them to use MRI to diagnose other computers with broken disk controllers. They conclude that the disk controller plays a key role in mediating disk access, and this is confirmed with a statistical mediation analysis. Someone even develops the technique of Directional Trunk Imaging (DTI) to characterize the structure of the ribbon cables (fiber tract) from the disk controller to the hard disk, and the results match the functional correlations between the hard disk and disk controller. But for all this, have they really understood how the computer works?

The neurophysiologist spoke up. “Listen here,” he said. “You have found the larger patterns, but you don't know what the individual circuits are doing.” He then probes individual circuit points within the computer, measuring the time course of the voltage. After meticulously advancing a very fine electrode in 10 micron increments through the hard material (*dura mater*) covering the CPU, he finds a voltage. The particular region shows brief “bursts” of positive voltage when the CPU is carrying out math operations. As this is the math co-processor unit (unbeknownst to the neurophysiologist), the particular circuit path is only active when a certain bit of a floating point representation is active. With careful observation, the neurophysiologist identifies this “cell” as responding stochastically when certain numbers are presented for computation. The cell therefore has a relatively broad but weak receptive field for certain numbers. Similar investigations of nearby regions of the CPU yield similar results, while antidromic stimulation reveals inputs from related number-representing regions. In the end, the neurophysiologist concludes that the cells in this particular CPU region have receptive fields that respond to different kinds of numbers, so this must be a number representation area.

Finally the neuropsychologist comes along. She argues (quite reasonably) that despite all of these findings of network interactions and voltage signals, we cannot infer that a given region is necessary without lesion studies. The neuropsychologist then gathers a hundred computers that have had hammer blows to various parts of the motherboard, extension cards, and disks. After testing their abilities extensively, she carefully selects just the few that have a specific problem with the video output. She finds that among computers that don't display video properly, there is an overlapping area of damage to the video card. This means of course that the video card is necessary for proper video monitor functioning. Other similar discoveries follow regarding the hard disks and the USB ports, and now we have a map of which regions are necessary for various functions. But for all of this, have the neuroscientists really understood how the computer works?

## The moral of the story

As the above tale illustrates, despite all of our current sophisticated methods, we in neuroscience are still in a kind of early stage of scientific endeavor; we continue to discover many effects but lack a proportionally strong standard model for understanding how they all derive from mechanistic principles. There are nonetheless many individual mathematical and computational neural models. The Hodgkin–Huxley equations (Hodgkin and Huxley, 1952), Integrate-and-fire model (Izhikevich, 2003), Genesis (Bower and Beeman, 1994), SPAUN (Eliasmith et al., 2012), and Blue Brain project (Markram, 2006) are only a few examples of the models, modeling toolkits, and frameworks available, besides many others more focused on particular phenomena. Still, there are many different kinds of neuroscience models, and even many different frameworks for modeling. This means that there is no one theoretical *lingua franca* against which to evaluate empirical results, or to generate new predictions. Instead, there is a patchwork of models that treat some phenomena, and large gaps where there are no models relevant to existing phenomena. The moral of the story is not that the brain is a computer. The moral of the story is twofold: first, that we sorely need a foundational mechanistic, computational framework to understand how the elements of the brain work together to form functional units and ultimately generate the complex cognitive behaviors we study. Second, it is not enough for models to exist—their premises and implications must be understood by those on the front lines of empirical research.

## The path forward

A more unified model shared by the community is not out of reach for neuroscience. Such exists in physics (e.g., the standard model), engineering (e.g., circuit theory), and chemistry. To move forward, we need to consider placing a similar level of value on theoretical neuroscience as for example the field of physics places on theoretical physics. We need to train neuroscientists and psychologists early in their careers in not just statistics, but also in mathematical and computational modeling, as well as dynamical systems theory and even engineering. Computational theories exist (Marr, 1982), and empirical neuroscience is advancing, but we need to develop the relationships between them. This is not to say that all neuroscientists should spend their time building computational models. Rather, every neuroscientist should at least possess literacy in modeling as no less important than, for example, anatomy. Our graduate programs generally need improvement on this front. For faculty, if one is in a soft money position or on the tenure clock and cannot afford the time to learn or develop theories, then why not collaborate with someone who can? If we really care about the question of how the brain works, we must not delude ourselves into thinking that simply collecting more empirical results will automatically tell us how the brain works any more than measuring the heat coming from computer parts will tell us how the computer works. Instead, our experiments should address the questions of what mechanisms might account for an effect, and how to test and falsify specific mechanistic hypotheses (Platt, 1964).

### Conflict of interest statement

The author declares that the research was conducted in the absence of any commercial or financial relationships that could be construed as a potential conflict of interest.
